# Providing holistic end-of-life care for people with a history of problem substance use: a mixed methods cohort study of interdisciplinary service provision and integrated care

**DOI:** 10.1186/s12904-024-01416-4

**Published:** 2024-04-01

**Authors:** Lucy Webb, Gemma Yarwood, Gary Witham, Sam Wright, Sarah Galvani

**Affiliations:** 1 Manchester Metropolitan University, Oxford Road, Manchester, UK; 2 Change Grow Live, 76 King Street, Orega, Manchester, UK

**Keywords:** Substance use, Homeless, Palliative care, Complex needs, End-of-life, Interdisciplinary care, Shared care, Integrated care

## Abstract

Harmful use of illicit drugs and/or alcohol is linked to life-limiting illness and complex health and social care needs, but people who use substances and have complex needs do not receive timely palliative care and fail to achieve quality standards for a good death. They and their families often require support from multiple health and social care services which are shown to be poorly integrated and fail to deliver interdisciplinary care. This study aimed to identify the existing barriers and facilitators within and between services in providing this population with a good death. Using a mixed methods approach of survey, focus groups and semi-structured interviews, we explored the perspectives of practitioner and management staff across a range of health and social disciplines and organisations in one combined authority in a large city in the north west of England. Our findings indicate that practitioners want to provide better care for this client group, but face structural, organisational and professional boundary barriers to delivering integrated and shared care. Differences in philosophy of care, piecemeal commissioning and funding of services, and regulatory frameworks for different services, lead to poor and inequitable access to health and social care services. Ways forward for improving care are suggested as bespoke hostel-based accommodation for palliative care for this client group, and specialist link workers who can transcend professional and organisational boundaries to support co-ordination of services and support. We conclude that it is no longer adequate to call for more training, better communication and improved joint working. Complex care at the end of life requires creative and cohesive systemic responses that enable multi-disciplinary practitioners to provide the care they wish to give and enables individuals using substances to get the respect and quality service they deserve.

**Supplementary Information:**

The online version contains supplementary material available at 10.1186/s12904-024-01416-4.

## Background

There is a reported dearth of evidence relating to palliative or end-of-life care specifically for people with problem substance use and life-limiting illness [1], but an increasing need in the United Kingdom (UK) associated with an ageing heroin-using population [2], and an increase in premature deaths from alcohol-related liver disease and hepatitis [3]. Indeed, Verma et al. [4] report that only approximately 30% of patients with advanced liver disease (approximately 80% being alcohol-related) have a referral to specialist palliative care, and these referrals are commonly in the last few days of life.

Alcohol use is associated with life-limiting chronic diseases beyond liver disease, including cancers, cardiovascular disease, and mental ill health, and, indirectly, type 2 diabetes, dementia, hypertension and stroke [5]. Use of illicit drugs such as heroin, cocaine and crack cocaine, cannabis, and amphetamines is also associated with cardiovascular disease, dementia, stroke and mental illness, but also blood borne viruses such as Hepatitis, HIV, and chronic obstructive pulmonary disease (COPD) due to the routes of administration, or sexually transmitted diseases and tuberculosis associated with lifestyle [6].

There is also an identified gap in the evidence that demonstrates effective service provision for this client group in specialist palliative care. Ebenau et al. [7] found care pathways and networks for this client group to be ineffective, as staff in different services fail to understand the needs of these clients, demonstrate poor communication, struggle to manage pain and do not support informal caregivers effectively. This results in delayed and poor care across a range of needs.

These clients often present co-morbid physical and psychological needs but also have problems with finance, housing, family dynamics and, often, involvement with the criminal justice system [8, 9]. They may also face marginalisation, stigma and even self-stigma, leading to social exclusion, late presentation to health services and consequent delayed treatment and support [10, 11].

Our work with this client group to date has shown problems with integrated care provision, particularly for vulnerably housed substance users with life-limiting illness [12]. This has also been shown to be problematic for homeless clients using hostel accommodation [13]. Integrated palliative care models exist for closer working between health specialisms [14, 15] but care across NHS, social care and voluntary sector providers is largely absent and particularly problematic for this population [11, 16–19]. Integrated Care Systems (ICSs) [20] aim to integrate health and care services within geographical areas and be based on local need. Evidence suggests however that those with complex needs experience lack of between-service co-ordination, and patient experience is not being taken into account when planning pathways [21]. Complex medical, social and behavioural needs require agencies to share knowledge and skills to deliver care that is appropriate for the individual and carers, particularly for those with vulnerabilities such as poor housing, mental ill health or complex needs. But we have found that stigma, marginalization and lack of organizational resources for this client group limit early identification of palliative status which results in late presentation and limited time to plan a good death [10, 11].

In the UK, substance use and housing is often commissioned from voluntary sector non-governmental organizations, presenting a structured divide between statutory and voluntary/commissioned services. Templeton et al. [22] identified how voluntary sector staff working in hostels have good skills in risk management and behavioural challenge for substance users, but struggle to manage those with palliative needs. They found hostel and community health staff had anxieties about managing controlled drugs on their own premises or in clients’ homes when working with substance users. Further challenges arise from silo working between health, social care, housing and palliative care agencies that creates gaps in pathways and limits care co-ordination for many service users with complex health and social care needs [23, 24].

The problem is exacerbated by gaps in policy frameworks. Currently, substance use receives limited attention in national or local policy and practice around end-of-life care (EoLC) in the UK and elsewhere, and EoLC receives little attention in national or local policy and practice around substance use. In the UK, this gap results in care provision that focusses on either substance use or EoLC, resulting in services and staff who are not equipped to respond adequately to these individuals, their family or friends [11].

## Methods

This study was part of a NIHR-funded project (NIHR 200054) to develop a model of care to maximize integrated working across sectors to deliver a good death for substance users with life-limiting illness. This paper reports on the mixed methods investigation of existing barriers to integrated care and effective service delivery for this client group, carried out in a large city in the North-West of England. The study involved ten partnerships with voluntary sector palliative care providers, homeless charities, and partners from primary care, secondary care, specialist palliative care teams, and local authority social services.

### Aims and objectives

The project aim was to determine what an improved model of care would look like for this population when delivering planned end-of-life wraparound care package, meeting holistic needs, enabling early intervention, supporting carers and empowering staff. This paper reports on the aim to identify the existing barriers and facilitators within and between services in providing this population with a good death.

The objectives for this project element were:

1. Identify the barriers and facilitators of service provision between and within services,
2. Identify the development needs that would better facilitate integrated care.
3. Gain understanding of systemic challenges to implementing improved integrated care.

We adopted a mixed methods approach to gain both measurable and informative data in order to identify the key service delivery issues for clients and staff, and gain an in-depth understanding of the ways the issues impact on clients and staff.

## Quantitative data collection and analysis

### Participants

Participants were frontline and management staff working in the 14 care settings and different NHS Trusts across one combined authority in the north west of England, comprising statutory and voluntary sector substance use services, adult social care, primary and secondary healthcare, housing charities, statutory and voluntary sector specialist palliative care providers (see Table 1).

### Tools

For the quantitative strand of the project, we developed bespoke questionnaires based on the Palliative Care Outcomes Scales (POS) [25]. A range of validated questionnaires were investigated for suitability for this project strand, however, all were either heavily focused on symptom control or service quality, without addressing the specific factors of service accessibility and quality of integrated care that were key outcomes for our project.

The bespoke questionnaire (17 items on a 5-point Likert scale, see supplement 1) retained the domain questions measured by the POS: *Physical, Psychological, Emotional, Spiritual, Information Provision* and *Support*. However, we added items to capture ease of access and inter-disciplinary service working and collapsed psychological, emotional and spiritual domains. The final version comprised of service and demographic descriptors, and rating items within six domains: Support given, Ease of Access, Information provided, Interdisciplinarity, Psycho-socio-spiritual, Physical.

A normality test was performed on each domain (Physical, Psychosocio-spiritual, Support, Information provision, Inter-service working, Access). All domains showed significance by the Shapiro-Wilk test, indicating non-normal distribution. Individual items were also tested for normal distribution, all demonstrating skewness or kurtosis, indicative of significant findings from the survey’s Likert scales.

### Procedure

The survey tools were uploaded to Qualtrics™ online survey platforms, staff from the 14 partner agencies were invited to complete the survey if they had experience of delivering care to a client with both problem substance use and a life-limiting illness at the stage of being or becoming palliative.

## Qualitative data collection and analysis

The qualitative data collection sought to complement the quantitative data by documenting, in greater depth, the experience of practitioners who cared for someone who used substances and had palliative or end-of-life care needs.

Practitioner evidence was collected through four focus groups with front line practitioners (*n* = 24) and semi-structured interviews with managerial level staff (*n* = 13) across EoLC services, substance use services, housing and community organisations. The aim was to explore their views and experiences of current practice and service provision, identify strengths and weaknesses, and consider how to improve practice. The development of the focus group topic guide and the semi-structured interview schedule (see supplements 2 & 3) were informed by several sources: (i) findings from our previous exploratory research project [11], (ii) input from our advisory group comprising people with lived experience, and (iii) participatory workshops held earlier in the project to facilitate the development of a theory of change and associated model of care.

Positioning analysis was used as an analytical framework [26] enabling exploration of how the participant positions their own actions and interactions, and how those positions relate to a broader normative cultural framing of such interactions [27]. Positioning occurs in dialogue and as such is a discursive process. Underpinned by Positioning Theory, the analysis explores the construction(s) (or attempted construction(s)) of action and highlights the explicit and implicit patterns of reasoning that stem from the ways that people act towards each other. In doing so, positioning analysis enables a critical framing of interaction, for example, where participants communicate from multiple standpoints, rather than through unequivocal identities and norms [28].

The procedure for analysis, supported by Nvivo 11 software, involved:

1. Listening twice to the focus group recordings.
2. Systematic reading of each transcript.
3. Annotations highlighting the positionings of the participants.
4. Identifying common constructions and positions across all four focus groups.

This process generated common underlying themes. For quality control purposes this was led by one researcher and reviewed independently by a second.

Template analysis [29] was the method adopted for analysis of the semi-structured interviews. This method allows for *a priori* themes to be identified in the data while also allowing for identification of emergent themes. The process for analysis is:

1. Reading of a sample of transcripts.
2. Development of draft thematic template.
3. Grounded coding of all transcripts.
4. Amending of/additions to thematic template.

This is a transparent and honest form of data analysis. It enables new themes to be identified while simultaneously reflecting that other themes would be highly likely to ‘emerge’ from the data given the direction of the interview questions.

## Ethics

Ethical approval for the research was gained through the UK’s Health Research Authority approval process (REC reference 20/WM/0140). Additional approval was required by some of the 10 partner agencies.

## Results

### Quantitative results

#### Survey findings

A total of 117 responses were obtained from participants from 14 care settings in the North West of England, reduced to 98 following adjustments for missing values. Thirty respondents worked in social care (30%), 20 in voluntary or statutory palliative care (20%), 19 in addictions (19%), eight in primary care (8%), 12 worked for housing charities (12%), and one in mental health care (Table 1).

**Table 1:** Participants in quantitative survey

Role	Housing	Substance use	Palliative/EoLC	Primary care	2nd ary care (EoLC)	Adult social care	Mental health
Manager	3	6	1		1	7	
Qual. Practitioner (nurse, medical)	2	7	13	8	3	22	1
support worker	7	6	3				
Consultant (nurse, medic)			5		3		
Other/allied professional			1			1	
Totals	12	19	23	8	7	30	1

Of those who responded to the specific question on their clients’ main presenting problem (96), 28% identified physical or mental health problems as the main problem, 25% described social issues as the clients’ main problem, 24% reported palliative care as the clients’ main problem, and 19 (18%) identified addiction as the main problem.

#### Domains

Analysis of items within each domain was conducted to gain a breakdown of specific needs in order to identify unmet need. Items for each of the six domains (physical, psycho-socio-spiritual, support, information provision, inter-service working, access to palliative care services) were scored, with lower scores representing less problem. Domains with 95% confidence intervals either above or below the central score (2.0) demonstrate definitive findings (Table 2).

The physical domain results indicate that care staff in general rated the physical needs of clients as most likely to present with unmet need, and the psycho-socio-spiritual domain was shown to be the second most likely to present with unmet need. Types of physical issues were reported by 75 respondents as withdrawal from substance use (32%), pain (28%) and respiratory problems (21%). Ease of access, information and support given to these clients and their families were rated well by staff members generally. Interdisciplinarity was not rated as either particularly effective or as presenting a problem for staff.

**Table 2:** Mean scores for questionnaire domains (> 2 = perceived problem)

Domain	Mean (range 0–4)	95% CI
Physical	2.62	2.44–2.78
Psycho-socio-spiritual	2.37	2.21–2.54
Information provided	1.34	1.13–1.55
Interdisciplinarity	2.05	1.84–2.26
Ease of access	1.52	1.26–1.78
Support given	1.33	1.10–1.56

### Comparison of job roles

A comparison of ratings by staff role/service provider reveals different perceptions of the domains by service. Staff in different roles show contrasting perceptions of domains, whereas, when treated together as above, these differences average out (regress to the mean).

#### Interdisciplinarity

When interdisciplinarity was analysed by staff role, a Kruskal-Wallis H test showed that there was a statistically significant rating difference in interservice functioning between job categories (χ^2^(2) = 10.042, *p* = 0.018), with a mean rank interservice score of 45.79 for palliative care, 40.98 for social care, 36.31 for addiction care and 21.75 for housing care, indicating that palliative care staff rated interservice functioning as most problematic for their clients. This counters the overall mean scores for the domains suggesting that other health care staff (secondary and primary care) rated interservice functioning as effective.

#### Access to palliative care

Access to palliative care was rated most problematic by social care staff (Kruskal-Wallis H: χ^2^(2) = 17.627, *p* < 0.001, mean rank 41.10), in comparison with the palliative care staff rating (mean rank 35.46), addiction care staff rating (mean rank 25.89), and housing services staff rating (mean rank 16.14). while this is clearly the practitioners’ perception rather that of the client or their family, the difference between professions is significant.

#### Psycho-socio-spiritual care

Social care staff also rated psycho-socio-spiritual problems for their clients significantly higher than addiction, housing or palliative care colleagues (χ^2^(2) = 10.999, *p* = 0.012) with a mean rank psycho-socio-spiritual score of 41.23 in comparison with the addiction mean rank of 29.15, the palliative care mean rank 24.71 and the housing mean rank 22.88.

#### Physical care and information-giving

There was no significant difference between service staff rating for information provided or physical problems for their patients/clients. Numbers were too small to calculate findings directly from staff from other primary care and secondary care, however, such data is considered in its influence on the findings. Therefore, services most involved are likely to be finding physical care most challenging, as identified in Table 2.

#### Support given

For practical support given, i.e., additional support for social and family needs, there were no significant differences between staff ratings by service type, and the mean scores for all services indicates that all rated this domain as not presenting a problem or unmet need (Table 1).

### Qualitative findings

#### Focus groups

Table 3 below outlines the characteristics of the 24 participants in the focus groups:

**Table 3:** Focus group sample sizes and group characteristics

Focus Group	Participant numbers	Practitioner Role
Focus Group 1	7	Social worker (*n* = 3)
		Hostel manager (*n* = 3)
		Regional palliative care manager (*n* = 1)
Focus Group 2	5	Social worker (*n* = 2)
		Palliative care registrar (*n* = 1)
		Hostel manager/worker (*n* = 2)
Focus Group 3	8	Social worker (*n* = 3)
		Hospice nurse (*n* = 1)
		General Practitioner (GP) (*n* = 1)
		Substance misuse worker (*n* = 3)
Focus Group 4	4	Hostel manager/worker (*n* = 2)
		Hospice nurse (*n* = 2)
**Total**		**24 people**

The following themes were generated from these data:

#### Professional boundaries and service development needs

There was a separation between “health” and “social care” with case study exemplars given by focus group members. These highlighted the gaps in service and different viewpoints about where the responsibilities lie in supporting people using substances at the end of life. Participants agreed that this population often required complex care and that trying to coordinate multiple services in a person-centred approach was challenging. However, social workers emphasized personal care and medication as a missing need from their service perspective, while themselves focusing on housing, and health practitioners identified socio-economic issues as unmet need.

#### Maintaining moral adequacy in the face of traumatic death

One participant working within hostel and temporary housing presented the dilemma of clients having to leave for more suitable accommodation and although this was facilitated to support care and positioned by the participant as an ethical justification. The client, however:

…felt like he was being kicked out through no fault of his own, [ ] but we knew in the long run that that service was more beneficial for him, and he could get the care and support that he needed (hostel worker 1)

This raises the dilemma that social care staff working with such clients have built up relationships that would be lost within a transfer to another service. Maintaining moral adequacy meant having conversations with service providers and advocating for clients:

…we feel like we’re forced into a position of questioning ourselves all the time, “Have we done enough?” [ ] because you’re forced between two services. So, what we’ve got is [ ] our service which is trying to report somebody who may be at end-of-life and ensuring that they’ve got the appropriate accommodation without us having the appropriate accommodation.” (hostel manager)

#### Working with a client/patient group where a “good” death is not necessarily their priority

There was a clear problem of understanding clients who prioritized their substances and lifestyles over how they were to die. Social care staff reported that accessing alcohol or substances and not physical self-care was a priority for many clients. So, for example, attending outpatient appointments was not a priority and this often led to a withdrawal of medical services based on perceived non-engagement. The social workers reported finding health-related services not responsive to the complex needs of this population and lacked understanding of the self-neglect and values of these clients. The salience of lifestyle over health presented problems in placing clients when in need of palliative care. For one social worker trying to locate an appropriate smaller hostel was challenging when there was ongoing substance use. Continuing substance use also made access to palliative environments challenging as it causes significant problems managing symptoms. Hostel staff however understood the need to continue offering support, as one hostel manager described:

One of ours, not at all well, got COPD and other issues, has chosen the last.

two nights to sleep out and you’d think, well in the current weather.

conditions that’s fairly insane isn’t it? But we will just continue to say, “Your

door’s open, please return, is there anything that can keep you in, rather

than you going out?” (hostel manager)

#### Where’s an appropriate place to die?

Hospitals were acknowledged by all participants to be inappropriate places for these clients at end-of-life. This was for a number of reasons, with one senior hostel manager suggesting it was the challenges of getting to know the client since they often did not want to engage with health-initiated services.

There was an acknowledgement, particularly presented by social care practitioners, that stigma and discrimination was more explicit within health-led services, and that this affected client engagement. A social worker commented:

They’re still people after all, and they’ve got the right to be able to choose.

how they wish to end their life, whether they want to be at home. A lot of.

people are afraid to go into hospice care, into hospital care, because they.

feel that they won’t be able to drink, so they often will put themselves at.

more harm really. (Social worker)

Some participants understand that the behaviour of this client group can be a challenge for health-related services, but even some homeless charities refuse entry to people using drugs or alcohol. From a palliative care perspective, hospices were not seen as a long-term place of care and, if the person using substances improved, they would seek to discharge the person from a hospice.

#### Semi-structured interview findings

Table 4 below outlines the characteristics of the 13 practitioners who participated in the semi-structured interviews.

**Table 4:** Participant profiles: role, sector, geographical remit (HC = health care, SC = social care, SCHC = dual role)

Palliative care		
1. Consultant in Palliative Medicine	Statutory sector - HC	Trust-wide hospital and community care
2. Consultant in Palliative Medicine; & Medical Director	Statutory and voluntary sectors - HC	Hospital (secondary care & voluntary sector palliative care organisation)
3. Clinical nurse specialist in PC	Statutory sectors - HC	Primary care
4. Clinical nurse specialist in PC	Statutory sectors - HC	Primary care
5. Clinical nurse specialist in PC	Statutory sectors - HC	Primary care
**Substance use**		
6. GP & Medical Director	Voluntary sector - HCSC	Substance use organization
7. Specialist Team Manager – Community addiction Service	Statutory sector - SCHC	Substance use, mental health and physical health services
8. Team Manager – Community Drug and Alcohol Team	Statutory sector - SCHC	Substance use services
9. Community manager	Statutory sector - SC	Adult social care
10. Service manager	Vol sector – SCHC	Substance use organization
**Other community**		
11. Director of Homeless Services	Voluntary sector - SC	vulnerable and homeless population; population with complex needs; community services including families
12. Advanced Practitioner in Public Health	Statutory sector – SC	Substance use, blood borne viruses, sexual health
13. GP with special interest in alcohol and other drugs	Statutory sector- HC	Primary care, community

#### Current barriers to effective service responses

A variety of responses were received to questions about how services currently responded to people who were using substances while receiving palliative and end-of-life care. This variation was noticeable between disciplines but also within disciplines.

Among participants from hospital-based and community-based palliative care provision, the variation depended on their particular setting and work context. A hospital-based provider commented that one barrier in current models of care was the amount of different organisations to liaise with:

So, we have a separate hospital for people with heart and chest complaints, a separate hospital for people with neurology and neurosurgical complaints, a separate hospital for people that have got cancer problems, a separate hospital for people with women’s health and gynaecology problems and gynaecological oncology problems, a separate hospital for children. (PC Consultant)

Another barrier was the lack of questions about substance use within palliative care assessments. None of the palliative care respondents reported the inclusion of questions relating to substance use in their assessments in spite of additional considerations about opioid medication dosage, availability of medications, and ‘uptitrating’ someone with a history of opiate use to minimise their pain. A potential facilitator was the option of liaising with substance use services to sustain community prescribing with one participant suggesting they could expand their befriender service to support people with substance use issues.

As with the palliative care specialists, the substance use practitioners reported no routine questioning that could help detect life-limiting illness. They reported more general health questions with most offering further nurse-led health assessment if they had concerns. However, practice varied from one practitioner to the next. One person recounted talking to his client about his wishes at end-of-life and how his son could help him to sort his collection of books. They also managed to discuss naloxone administration with his son. However, the lack of routine questioning suggests early (or any) intervention may be missed.

The practice of one substance use service was to withdraw service provision and prescribing services if the person was receiving palliative or end-of-life care, although they would continue to advise palliative care nurses who might be nervous about supporting people using substances. While this may avoid double prescribing, it may miss the opportunity for continued substance-related care at end-of-life, particularly if people became non-verbal.

One of the barriers faced by substance use professionals was knowing when someone was end-of-life as people’s health conditions fluctuated and that fluctuation could reflect their recent use of, or lack of, substances. In terms of good practice, one service had introduced a monthly death review panel comprising the medical lead, keyworker and team leader. They also attended the quarterly multi-agency and multi-disciplinary death review panels held across two counties in the region.

Another barrier was the availability of local resources as these varied from location to location. In one Borough that was split in two, one had a community palliative care service and the other did not, resulting in a referral back to the GP or to other specialists. This results in the person needing to make yet another appointment with another service rather than being able to be ‘held’ in one service supported by specialist advice. Adult social care involvement was also difficult to secure unless there was a clear safeguarding issue or they were attached to a mental health team.

A key barrier reported by one social care manager was the lack of a well-developed pathway resulting in a poor response from other services. While they were attempting to improve joint working with local hospices they stated that they ‘weren’t there yet’.

More positively, one housing organisation had a good partnership working with a specialist primary health care team locally and enabled them to balance what the person needed medically with the person’s wishes for accommodation and support approaching end-of-life. This service also set up ‘in-depth debrief’ following a person’s death in their service to ensure their practice was as good as it could be.

For all specialists, there was complete agreement about the need for better joint working. It was variously described as inadequate, uninformed, disjointed, untimely, and lacking a needs-led approach. Gaps between mental and physical health teams and between community palliative care and community drug teams were identified as particular barriers, although intra-organizational multi-disciplinary working was also identified as lacking. As one participant stated: “A better MDT approach would mean better outcome” (Senior health professional).

One senior health professional summed up the frustration many of the participants felt:

How do you get that sense that we’re all working collectively together as a team even though your badges are different? How do you move beyond the badge to get that sense of team and identity? … How do you get beyond old practices of working - to try and create that? And recognise safety and risk on a wider patch to help people so that you think, ‘Well I’m all right Jack’ but actually when that person moves on into being primarily under another service, or is actually under three or four people because of the problem that they have, how do you bring things together? (Senior health professional)

One participant from a substance use service said there was a need for people to have “stability and consistency” but added that, given the lack of stability and consistency in services, this was a considerable challenge.

In addition to the lack of knowledge about resources and where to refer individuals and families, there was a reported lack of communication across and between specialist providers. For example, where someone had a liver, renal, and lung specialist all involved in their care, all information was returned to the GP without any ‘real MDT working’. One participant felt that national policy focus on recovery had resulted in a lack of clinical expertise in substance use services – a lost skillset when it comes to physical health and illness - that would be important for this group of people.

#### Gaps in services

As this is a new area of research and practice consideration, it is not surprising that gaps were identified in terms of processes, resources and service provision. One palliative care practitioner stated: “There are almost too many gaps to quantify.” Chief among these gaps, and with agreement across all participants’ specialist areas, was the need for accommodation and/or bed space that is suited to the needs of people continuing to use substances approaching the end of their lives. One participant described the provision of that bed space as “absolutely paramount” in order to offer care that meets their needs stating:

It’s that availability of being treated as humans as well, that’s the place of care, that’s definitely a barrier, we’ve struggled to get people into beds sometimes… (Senior health professional)

This was felt to be important because this client group would feel more comfortable in a more familiar environment (within a hostel), there would be no pressure to ‘abstain’ and being with a workforce that understood their behavioural choices.

There was a great deal of enthusiasm for a specialist role for supporting people across the range of services, acting as a bridging role between services. As a substance use manager stated, it would be helpful,

… if there were specialist workers who were the ones who integrated all the services together who were that conduit, … (Senior substance use professional)

Specific suggestions were for a palliative care link nurse to liaise between primary care, hospitals, palliative care, mental health and substance use services. Also suggested was a specialist homelessness link worker with a ‘foot in both camps’ of healthcare and homelessness to overcome barriers of care culture and develop pathways between hostels and health services. As one substance user worker stated:

I know people have often felt there could have been better liaison with hospitals who are discharging somebody who is end-of-life. … It’s not a criticism of any person, it’s actually, … you think, “Why didn’t you ring us up a couple of weeks – a month earlier?” There’s probably a good reason why they didn’t, and I’m sure they have similar frustrations, but with us. … it’s that liaising, it’s that joined-up work if you like, that often doesn’t take place as soon as it could have done to make things easier for the person who, at the end of the day, we’re all actually looking after, doing our bit. (Senior substance use professional)

## Discussion

### Quantitative findings

The main unmet need for clients, as reported by both social and health care staff in the survey, was their physical care. This is likely to be a concern among all staff for their clients in need of palliative care, however, these self-report ratings may also indicate staff members’ focus on physical care with less regard or awareness for issues such as access to palliative care or family needs. It is interesting to find that social care staff are most concerned with psycho-socio-spiritual care, in comparison with palliative and housing care staff who rate this issue as of least concern. This may reflect the job roles but also the frustration of care workers who may value and recognise this need but have fewer resources to address them. This may explain why social care staff rate this as an unmet need more highly than palliative care staff who have more relevant resources. This does not explain why housing staff do not rate this particularly as an unmet need, however, it is possible that practical housing issues and chaotic substance use may be seen as key priorities for these services.

Interdisciplinary functioning and ease of access were rated differently by staff in different job roles, with those in secondary and primary health care roles experiencing a degree of effectiveness and those in social care and palliative roles finding this a problem. This suggests that care pathways may be experienced as more effective in primary and secondary care, with good referral routes, while referring in to health care or across services at tertiary level is experienced as ineffective. With recent focus on integrated care and care pathways within the NHS, these findings may indicate where barriers remain within a whole systems approach to seamless care pathways, especially between health and social care. This is supported by the findings for access to palliative care in which social care, addiction and housing staff experience this as significantly problematic.

Overall, there is a difference in perception of effectiveness of interdisciplinary working and access to palliative care by job role/type of service provider. It is likely that these findings are linked, representing a degree of silo working and limited or restricted care pathways that are not experienced by mainstream health services.

### Qualitative findings

Key qualitative findings demonstrate how the complex needs of people using substances at end-of-life are poorly served by professional boundary concerns and the lack of cross-agency working. There was also felt a lack of suitable training for staff, and the lack of client accommodation, particularly in relation to place of death, creating moral challenges for service providers. Practitioners were aware that the best care option for someone was not always what the client or their relatives wanted because of the limited social or community options. As is expressed by most people about place of death, the preferred option is to die at home [30], and this population is no exception. Our previous findings indicate that familiarity of surroundings, regardless of current homelessness or unstable housing, is still important for end-of-life [31].

Interviewees spoke of working together for a common goal asking how that might be done and identifying the need for a Community of Practice to help each other understand how people are ‘talking about ’people using substances approaching the end of their lives. Senior managers and clinicians across disciplines and agencies found points of disparity and points of agreement which illustrated their different perspectives and values. However, they largely all reported a lack of care pathways between agencies, and current responses to referral or joint working were limited overall and hugely variable according to service and staff member’s experience. They also noted that commissioning affected what could be offered given some areas had more services than others. They all agreed, however, that there was more to learn and to do.

### Synthesis of findings

Both sets of evidence indicate barriers to interdisciplinary working and shared care across services for these clients. Social care and voluntary sector services had most difficulty in accessing support or collaboration from health services. This has been identified as a communication problem created by lack of information-sharing agreements between statutory health services and commissioned voluntary sector services such as substance use or housing [32] and fragmented services [13]. Integrated care systems in England will need to tackle this issue for all statutory/voluntary sector organizations that overlap health services in order to improve inter-agency working. Commissioning, funding and role boundaries also present barriers to a systematized approach to shared care, as practitioners lacked confidence in extending their role to tackle complex care outside their remit or training, but lacked avenues to work with those that had such a remit. What appears to result is a ping-pong approach to meeting someone’s needs, with different agencies, practitioners and services dealing with individual needs, and care pathways that become tortuously long.

A key issue is shown to be a fundamental difference in philosophy of care between substance use service providers and palliative care, and medical treatment approaches and palliative care. Primary and secondary health and substance use services are currently focused on early detection of problem use for promotion of recovery, increase in social capital, and care pathways into treatment [33]. The medical treatment approach is also delivered as person-centred shared care rather than the substance use focus on behavioural motivation and change [7]. Neither approach is likely to fit with end-of-life and a focus on a good death.

This presents further challenge to the UK’s policy of integrated care. The different perspectives of care between disciplines, and perceived shortcomings of ‘other’ services, suggests a potential for blaming other services and restrictive practice. In general, integrated care may require a fundamental change in training for health and social care services to increase understanding of different philosophies, aims and values between disciplines. Indeed, this may need to extend to key performance indicators that currently remain bound within service functioning rather than lying with the patient/client needs.

A further concern emerging from both sets of data is the difference in defining or valuing ‘a good death’. Meier et al. [34] identified the three most important priorities for a ‘good death’ were preferences (i.e., where, who with), pain relief and emotional wellbeing. Additionally, Morgan and Gazarian [35] included opportunities for preparation and awareness. Our findings indicate divergence among disciplines as a result of different practice priorities, with substance use and social services not able to support preparation of the dying process, and health services not valuing emotional needs and lifestyle choice as much as symptom control. It is a concern that health and palliative care services may not recognise the effect that self-stigma may have on self-neglect and lateness of presentation.

Focus recently on liver disease pathways for early detection and treatment [36] offers a path to palliative care, but struggles to identify those not in contact with health services. Referrals from primary and secondary care through gastro-hepatology risk creating a lengthy pathway to end-of-life care. Service users and their families from our previous study [31, 37] reported that multiple contacts and appointments are difficult to manage and present yet another burden clients and their families need to navigate, so a long and multi-stage care pathway adds to this burden.

The context for lack of structure to the care process for this client group is arguably a decade of cuts to substance use services and many rounds of re-tendering and changing service providers. The English health and care systems have recently merged but are experiencing problems with joint working. Health providers commission many social care services from the non-governmental organization sector, particularly substance use services. Our findings reflect the disjointed nature of separate specialties, organizations, short-term commissioning and governmental strategies.

## Recommendations

This study identified key barriers to improving EoLC care for vulnerably housed substance users. We have summarized the key points that could improve services in Table 5.

**Table 5:** Key points for improving services

Problem identified	System change recommendation
Burden on client and family from multiple professional contacts	Link worker as a single point of contact to help navigate between services. Will provide advice, training, support and referral routes
Lack of early identification of palliative need and poor understanding of client reluctance and lifestyle	Community of Practice between services
Divergence of values and philosophies between services	Key performance indicators based on client needs, not service function
Extensive routes to palliative care	Clear pathways options, supported by link workers and community of practice
Limited community options for care and place of death preference not available	Hostel and housing provision to support those in palliative care; bespoke hostel/care accommodation
Fragmented services and poor inter-agency communication	longer-term service commissioning to establish lasting care pathways and networks. Integrated Care Systems to include voluntary sector organisations, with shared information and joint working

## Conclusion

It is axiomatic that inequalities in the delivery of palliative and end-of-life care could be improved by overcoming the barriers at individual, organisational, and structural/systemic levels. However, the first step is understanding what these different perspectives are and whether the systems and structures within which they sit help or hinder the effort to improve inequitable care. While systems and structures remain separated into health, social care and ‘others’, it is difficult to see how to overcome the professional boundaries that offer protection to the individuals and teams working in a minimally resourced environment. It is no longer adequate to call for more training, better communication and improved joint working. History tells us this clearly does not work and serves to pressure and blame individual practitioners. While integrated care is the current policy ‘ask’ in the UK, it is clearly not working for this group of people. Complex care at end of life requires creative and cohesive systemic responses. We therefore call for service commissioning within integrated systems to address the necessary multi-agency approach required for this client group and consider more creative planning to accommodate their needs.

### Electronic supplementary material

Below is the link to the electronic supplementary material.

Supplementary Material 1

Supplementary Material 2

Supplementary Material 3

## Data Availability

All electronic data was stored on a secure password-controlled drive, and retained for 10 years after publication of the results. Available from the author at reasonable request.Full study report is available at: https://e-space.mmu.ac.uk/631610.
