# Supplementary material for: Providing holistic end-of-life care for people with a history of problem substance use: a mixed methods cohort study of interdisciplinary service provision and integrated care
Source: BMC Palliat Care. 2024 Apr 1;23:86. doi: 10.1186/s12904-024-01416-4 (PMC10983728; doi:10.1186/s12904-024-01416-4)
Supplement: Supplementary file 1 — Supplementary Material 1 [file 12904_2024_1416_MOESM1_ESM.pdf]

Palliative Care Outcome Scale STAFF QUESTIONNAIRE (version 2)

Assessment no: ……………..

Staff code: ………………………...   Assessment date: ....

Date of birth: ..……………………………..………………...

Job title/role   ………………………………………………………………..

Care setting or service being described/experienced: (forced choice categories
Other … please state…..

………………………………………

Length of time in this service (in years)
………………………………………………………

What proportion of your client group do you estimate to have a life-limiting illness and use substances?   (sliding scale)

What proportion of your working week do you have direct contact with clients? (sliding scale).

Please answer the following questions about **your clients who may have both a life-limiting illness and a history of substance use** by ticking the box next to the answer that is most true for you. Thank you.

i) What is the main health or social care need for the clients you work with?

....

Palliative Care Outcome Scale STAFF QUESTIONNAIRE (version 2)

1. Access to services: how easy has it been in the last 2 weeks for your clients with life-limiting illness and substance use histories to get access to the service you work in?

- ❑ 0 Very easy
- ❑ 1 Easy
- ❑ 2 Neither easy nor hard
- ❑ 3 Hard
- ❑ 4 These clients have not been able to access the service

2. How would you rate the ability of your service in the last 2 weeks to work with other services for clients with life-limiting illness and substance use histories?

- ❑ 0 Very good
- ❑ 1 Good
- ❑ 2 Fair
- ❑ 3 Poor
- ❑ 4 They do not work with each other

3. Over the past 2 weeks, how much do you feel these clients generally have their time wasted by appointments at your service?

- ❑ 0 None at all
- ❑ 2 Up to half a day wasted
- ❑ 4 More than half a day wasted

4. Over the past 2 weeks, generally speaking, have practical matters relating to these clients' problems, either financial or personal, been addressed?

- ❑ 0 Practical problems have been addressed and their affairs are as up-to-date as I would wish
- ❑ 2 Practical problems are in the process of being addressed
- ❑ 4 Practical problems exist which were not addressed
- ❑ 0 They have had no practical problems

5. Thinking about a major concern that these clients often present with but that your service does not specialise in, how well does your service address this concern? This might be serious illness, substance use, housing or financial problems for instance.

Please say what this major concern is: ………………………………..

- ❑ 0 All the time
- ❑ 1 Most of the time
- ❑ 2 Sometimes
- ❑ 3 Occasionally
- ❑ 4 Not at all

6. Over the past 2 weeks, what one main physical symptom has concerned your clients with life-limiting illness and substance use histories the most. (i.e. pain, withdrawal symptoms, nausea, dizziness, etc)

symptom………………………………………………………………..

7. how much do you think your clients have been affected by this symptom? Please state what one symptom tends to concern these clients most (i.e. pain, withdrawal symptoms, nausea, dizziness, etc)

- ❑ 0 Not at all, no effect
- ❑ 1 Slightly - but not bothered to be rid of it
- ❑ 2 Moderately - symptom limits some activity
- ❑ 3 Severely - activities or concentration markedly affected
- ❑ 4 Overwhelmingly - unable to think of anything else

8. Over the past 2 weeks, have other symptoms generally been affecting how these clients feel?

Please say what these symptoms often are ……………………………….

- ❑ 0 No, not at all
- ❑ 1 Slightly
- ❑ 2 Moderately
- ❑ 3 Severely
- ❑ 4 Overwhelmingly

9. Over the past 2 weeks, have many of your clients with life-limiting illness and substance use histories have been feeling anxious or worried about their life-limiting illness or the care they receive for it?

- ❑ 0 No, not at all
- ❑ 1 Occasionally
- ❑ 2 Sometimes – it seems to affect their focus
- ❑ 3 Most of the time - often affects their focus
- ❑ 4 They often can't think of anything else - completely pre-occupied by worry and anxiety

10. Over the past 2 weeks, have these clients' families or friends been especially anxious or worried about them?

- ❑ 0 No, not at all
- ❑ 1 Occasionally
- ❑ 2 Sometimes – it seems to affect their focus
- ❑ 3 Most of the time - often affects their focus
- ❑ 4 They often can't think of anything else - completely pre-occupied by worry and anxiety

12. Over the past 2 weeks, generally speaking, how much information about the individual care they receive from your service has been given to these clients and their families or friends?

- ❑ 0 Full information or as much as wanted – always feel free to ask
- ❑ 1 Information given but can be hard to understand
- ❑ 2 Information given on request but they would have liked more
- ❑ 3 Very little given and some questions were avoided by staff
- ❑ 4 None at all – only when they demanded information

13. Over the past 2 weeks, have many of these clients been able to share how they are feeling with their family or friends?

- ❑ 0 Yes, as much as they wanted to
- ❑ 1 Most of the time
- ❑ 2 Sometimes
- ❑ 3 Occasionally
- ❑ 4 No, not at all with anyone

14. Family support services – over the last 2 weeks, how often have these clients' families or friends been supported/included by your service?

- ❑ 0 Yes, all the time
- ❑ 1 Most of the time
- ❑ 2 Sometimes
- ❑ 3 Occasionally
- ❑ 4 No, not at all

15. Over the past 2 weeks, do you think these clients generally have felt that life was worth living?

- ❑ 0 Yes, all the time
- ❑ 1 Most of the time
- ❑ 2 Sometimes
- ❑ 3 Occasionally
- ❑ 4 No, not at all

16. Over the past 2 weeks, do you think these clients have generally felt good about themselves?

- ❑ 0 Yes, all the time
- ❑ 1 Most of the time
- ❑ 2 Sometimes
- ❑ 3 Occasionally
- ❑ 4 No, not at all

17 Please tick which of the following best describes most of your clients:

- ❑ 0 Fully active
- ❑ 1 Restricted
- ❑ 2 Ambulatory
- ❑ 3 Limited self-care
- ❑ 4 Completely disabled

18. Please describe the extent to which your current service provision differs from what was provided prior to Covid 19.

…………………………………………………..

Categorised as:

1 Less face to face contact
2 restricted access to family
3 no significant change
4 staff shortages
5 improved MDT working
6 covid + have less access
7 PPE reduces relating
8 Reduced access/gatekeeping
9 Improved family & pt contact using phones
10 Increased hostel working
11 Reduced day care/group activities
12 Increased waiting lists

Any other comments

………………………………………………………..
