# Supplementary material for: Providing holistic end-of-life care for people with a history of problem substance use: a mixed methods cohort study of interdisciplinary service provision and integrated care
Source: BMC Palliat Care. 2024 Apr 1;23:86. doi: 10.1186/s12904-024-01416-4 (PMC10983728; doi:10.1186/s12904-024-01416-4)
Supplement: Supplementary file 2 — Supplementary Material 2 [file 12904_2024_1416_MOESM2_ESM.pdf]

Improving palliative and end of life care for people using substances

T1 Practitioner/Manager individual interview schedule

Interview preamble

- Do you have any questions from your reading of the information sheet?
- We're interested in your views and experiences and we may touch on some sensitive issues, but you don't have to talk about anything that you don't feel comfortable discussing. So just say if there are any questions you don't want to answer.
- Let me know if you are unsure about any questions or want me to repeat them.
- We can stop the interview any time - just say if you need a break at any time.

Consent form

- Recording and confidentiality
- Limits to confidentiality: harm or risk of harm to yourself or another
- Happy with everything so far? Any questions for me before we begin?

1. What is your job title and what does that entail? Who does your organisation provide services to?

2. How does your service currently respond when someone who uses/has used substances has a terminal illness? *[PROBE for separate issues if necessary]*
    a. To what extent does this happen?
    b. What is your own experience of this?
    c. Is there any way this could be improved? *[PROMPT ie: conditions recognised as being terminal; groups of people recognised as approaching the end of their life]*
    d. To what extent is asking about substance use and terminal illness part of routine questioning and assessment for people entering the service? Who does this?
    e. What support is available in your service for family/carers?

3. What support have you/your staff had for working with this group of people?
    a. How knowledgeable do you feel you/your staff are in working with a) the person themselves or b) family carers in these contexts?
    b. How comfortable do you feel you/your staff are in working with a) the person themselves or b) family carers in these contexts?
    c. It can be difficult for practitioners to talk about EoL care and/or substance use – how can they best be supported to overcome this?
    d. What might help you/your staff feel more supported?

4. What do you think good quality end of life care looks like for people who use/have used substances?
    a. How could we improve local palliative / EoL care for people who use/have used substances?
    b. How could we improve it for family/carers?

5. How can we improve current systems or pathways to enhance care quality?
    a. Where are the gaps in current care delivery models, if any? How could they be addressed?
    b. [For managers] Thinking about commissioning processes, what is needed to facilitate filling these gaps?
    c. [For managers] What commissioning constraints exist? How can these constraints be overcome?
    d. If resources were no object what would be possible? [*Prompt if needed*]

6. In your experience, what barriers to accessing end of life care exist for people who use/have used substances and who currently have little or no involvement with services?
    a. Who is currently missing from EoLC provision?
    b. How can we improve access to services for these people? *[PROMPT: for both practice and policy/commissioning change]*
    c. How could a new model of care expand access to EoL or social care?
    d. What resources are available to support this work? How could any resource constraints be overcome?

7. To what extent is it possible currently to deliver joined-up care between health and social care services [*PROMPT if necessary for: substance use and end of life care services]*?
    a. How could a new model of care improve this?
    b. What support should be incorporated into a new model of care to help practitioners maximise joined-up working across substance use, eolc, health and social care services?
    c. Which other services need to be centrally involved in this work? And which ones are currently missing?

8. What sort of training have you and your staff had to date on working with people at end of life who use / have used substances?
    a. What sort of training do you think would be helpful for you, your staff and your organisation under a new model of care?
    b. What do you feel your staff need to know in order to work effectively in these situations?
    c. Do your staff have any training needs relating to assessment processes/routine questioning around substance use, end of life care or other support needs?

9. Any other comments you would like to make or information you would like to share?

Thank you
