# Supplementary material for: Providing holistic end-of-life care for people with a history of problem substance use: a mixed methods cohort study of interdisciplinary service provision and integrated care
Source: BMC Palliat Care. 2024 Apr 1;23:86. doi: 10.1186/s12904-024-01416-4 (PMC10983728; doi:10.1186/s12904-024-01416-4)
Supplement: Supplementary file 3 — Supplementary Material 3 [file 12904_2024_1416_MOESM3_ESM.pdf]

Improving palliative and end of life care for people using substances

T1 PRACTITIONER FOCUS GROUP TOPIC GUIDE

TOPIC GUIDE: [Sub questions are prompts]

INTRODUCTIONS: Do you all know each other?

We'd like to start by asking you to think about your experience of having worked with people with terminal illness who use/have used substances.

1. What is your organisation's current practice when someone who uses/has used substances has a terminal illness?
    a. To what extent does this happen?
    b. What is your organisation's experience in delivering this? *[PROMPT for any limitations]*
    c. What is your experience in practice?
    d. To what extent is asking about substance use and terminal illness part of routine questioning and assessment for people entering the service? Who does this?
    e. What support is available for family caregivers?

2. What support have you had for working with this group of people?
    a. How supported do you feel in working with a) the person themselves or b) family members in these contexts?
    b. What support do you need?
    c. It can be difficult for practitioners to talk about EoL care and/or substance use – how can they best be supported to overcome this?
    d. What might help you feel more supported?

3. What does good quality end of life care look like for people who use/have used substances?
    a. How could we improve local palliative / eol care for people who use/have used substances?
    b. How can we improve current systems or pathways to enhance care quality?
    c. What might a model of care look like locally in terms of palliative or eol care for people using substances?

   d. Where are the gaps in current care delivery models, if any? How could they be addressed?

   e. If resources were no object what might it look like? [*Prompt if needed*]

4. What barriers to accessing EoLC exist for people who use/have used substances and who currently have little or no involvement with services?

   a. Who is currently missing from EoLC provision?

   b. How can we improve access to services for these people? *[Prompt for both practice and policy/commissioning change]*

   c. How could a new model of care help those people to access eol or social care?
   d. What resources are available to support this work? How could any resource constraints be overcome?

5. To what extent is it possible currently to deliver joined up care between substance use and palliative / end of life care services?

   a. Why is that? What helps/hinders joined up care?

   b. How could a new model of care improve this?

   c. What support could be incorporated into a new model of care to help practitioners maximise joined up working across substance use, eolc, health and social care services?

   d. Which other services need to be centrally involved in this work? And which ones are currently missing?

6. What sort of training have you and your colleagues had to date on working with people at end of life who use/have used substances?
   a. What sort of training do you think would be helpful for you, your colleagues and your organisation under this new model?
   b. What do you feel you need to know in order to work effectively in these situations?
   c. Did the training cover the breadth of people who come into this group? Was that helpful?
   d. Do you have any training needs relating to assessment processes/routine questioning?

7. Any other comments you would like to make or information you would like to share?

Thank you
